# Handedness is a biomarker of variation in anal sex role behavior and Recalled Childhood Gender Nonconformity among gay men

**DOI:** 10.1371/journal.pone.0170241

**Published:** 2017-02-24

**Authors:** Ashlyn Swift-Gallant, Lindsay A. Coome, D. Ashley Monks, Doug P. VanderLaan

**Affiliations:** 1 Department of Psychology, University of Toronto, Toronto, Ontario, Canada; 2 Department of Psychology, University of Toronto at Mississauga, Mississauga, Ontario, Canada; 3 Child, Youth and Family Division, Underserved Populations Research Program, Centre for Addiction and Mental Health (CAMH), Toronto, Ontario, Canada; Brock University, CANADA

## Abstract

Developmental theories of the biological basis of sexual orientation suggest that sexually differentiated psychological and behavioural traits should be linked with sexual orientation. Subgroups of gay men delineated by anal sex roles differ according to at least one such trait: gender expression. The present study assessed the hypothesis that handedness, a biologically determined sexually differentiated trait, corresponds to differences in subgroups of gay men based on anal sex role. Furthermore, it assessed whether handedness mediates the association between gender nonconformity and male sexual orientation. Straight and gay men (*N* = 333) completed the Edinburgh Inventory of Handedness and the Recalled Childhood Gender Nonconformity Scale. Gay men also completed measures of anal sex role preference. As in previous studies, gay men showed greater non-right-handedness and gender nonconformity than straight men. Also, among gay men, bottoms/versatiles (i.e., gay men who take a receptive anal sex role, or who take on both a receptive and insertive anal sex role) were more gender-nonconforming than tops (i.e., gay men who take an insertive anal sex role). In support of the hypothesis, bottoms/versatiles were more non-right-handed than tops and handedness mediated the male sexual orientation and anal sex role differences in Recalled Childhood Gender Nonconformity. Together, these findings suggest that developmental processes linked to handedness underpin variation among men in sexual orientation and gender nonconformity as well as variation among subgroups of gay men that are delineated by anal sex roles.

## Introduction

Handedness, or predominantly using the left or right hand for various tasks, is a marker of cerebral lateralization (for review, see [1]). Extensive research suggests that handedness is influenced by genetic factors and is also linked to sexually dimorphic endocrine and immunological mechanisms [2–5]. Although the factors affecting handedness are still under investigation, there is consensus that it is determined prenatally by biological factors (for review, see [6]).

Given this prenatal, biological, basis of handedness, the higher prevalence of non-right-handedness among men compared to women suggests that it also marks prenatal developmental processes influencing brain sexual differentiation (for review, see [7]). As such, handedness has been examined in relation to within-sex variation in psychological and behavioral domains that show large sexual dimorphism to provide insight into whether mechanisms underpinning handedness also influence these domains. Namely, these domains include gender expression and sexual partner preference.

In both sexes, non-right-handedness has been associated with elevated gender nonconformity. Left-handed women are more male-typical on a gender conformity scale (i.e., Bem Sex Role Inventory: [8, 9]) and highly gender-nonconforming natal male children are more non-right-handed [10]. A higher prevalence of non-right-handedness has also been reported in male-to-female and female-to-male transsexuals, regardless of sexual orientation, compared to male and female controls [11–14]. Furthermore, gay men and lesbian women, who show elevated gender nonconformity [15], also show higher rates of non-right-handedness compared to straight men and women [16–18].

Of note, non-right-handedness appears to be a masculine trait in that it is more common among straight men than straight women, and among lesbian women than straight women. However, non-right-handedness is also more common among gay men—who are more feminine on average—than straight men. One possible explanation for this apparent paradox is that non-right-handedness has a non-linear association with sexual differentiation among males. Specifically, among straight men non-right-handedness might be associated with greater male-typical gender expression whereas among gay men it might be associated with greater female-typical gender expression. As such, across straight and gay men, one would predict a curvilinear association between handedness and gender (non)conformity.

Handedness has yet to be examined in relation to the variation in gender nonconformity that exists both between *and* within sexual orientation groups. Whereas a number of studies have shown sexual orientation differences in gender nonconformity, only more recently has quantitative research begun to identify behavioral correlates of variation in gender expression among subgroups of same-sex attracted individuals. Among gay men, variation in gender nonconformity appears to correspond with variation in anal sex role behavior. Specifically, gay men with an insertive anal sex role (i.e., tops) scored higher on masculine personality traits compared to males with a receptive (i.e., bottom) anal sex role preference (i.e., bottoms; [19]). Conversely, bottoms scored higher on feminine personality traits compared to tops. Consistent with these reports, Moskowitz and Hart [20] found that self-identified tops rated themselves as more masculine compared to bottoms, and Zheng, Hart, and Zheng [21] found that tops were more likely to score higher on male-typical cognitive styles while bottoms were higher on female-typical cognitive styles.

The present study examined the hypothesis that handedness—a trait that is putatively tied to prenatal brain development, including brain sexual differentiation—is associated with variation in gender nonconformity both between and within male sexual orientation groups. One prediction tested was that, across straight and gay men, a curvilinear association exists between handedness and gender (non)conformity such that non-right-handedness is associated with greater gender conformity among straight men and greater gender nonconformity among gay men. In addition, based on research indicating a relationship between bottom anal sex role with greater female-typicality, and top anal sex role with greater male-typicality, it was predicted that these groups should also differ in handedness, with those who report bottom anal sex role being more non-right-handed. Lastly, it was examined whether handedness mediates sexual orientation as well as anal sex role-related differences in gender nonconformity.

## Materials and methods

### Participants

Participants were recruited at the 2015 Toronto Pride Festival and on Facebook. At the Toronto Pride Festival, experimenters explained to festival attendees that they were researchers from the University of Toronto conducting a study on developmental theories of sexual orientation. Experimenters offered a business card with the survey’s website, and/or recorded their email addresses as a means to send the website address for our online survey. Facebook advertisements targeted men who indicated that they were interested in men or men who indicated that they were interested in women. Only those 18 years of age and older were targeted, and the advertisements were presented only to those who spoke English. The locations selected included countries in which English is a first language (i.e., Canada, United States, New Zealand, United Kingdom, Australia). We emailed details regarding how to complete our online questionnaire to 459 people from the Toronto Pride festival, and our Facebook advertisements reached 56,155 people. A total of 598 participants (56 from Toronto Pride and 542 from Facebook) completed > 90% of the items on our questionnaire. Participants were excluded from the present study if they did not report their age, sexual orientation or, in the case of gay men, their anal sex role preference. Of the 598 participants who completed > 90% of the questionnaire, 159 did not identify their sexual orientation and 208 did not report their age. Of the remaining participants, 242 gay men indicated their preferred anal sex role, and 222 also reported their anal sex role behavior. The questionnaire was hosted on Qualtrics using their survey software. The study was approved and conducted in accordance with the guidelines from the Human Research Protections Program at the University of Toronto. The letter of information and informed consent forms were presented to participants via Qualtrics prior to the questionnaire. Participants had to click a box indicating that they read the ethics statement and agreed to participate. Consent and questionnaire data were encrypted on a computer and password protected.

The final participant numbers were as follows for all analyses excluding the anal sex role behavior analyses: straight men: *n* = 91; all gay men: *n* = 242; bottoms: *n* = 91; versatiles: *n* = 115; tops: *n* = 36. For the analyses on anal sex role behavior, the total number of participants were as follows: straight men: *n* = 91; all gay men: *n* = 222; bottoms: *n* = 96; versatiles: *n* = 69; tops: *n* = 57. The mean (SD) age for straight men was 32.26 (15.23) years and 33.61 (13.47) years for gay men. The mean (SD) ages for anal sex role preference groups were as follows: bottoms were 33.27 (13.28), versatiles were 33.83 (13.65), and tops were 33.80 (13.77). The mean (SD) ages for anal sex role behavior groups were as follows: bottoms were 32.21 (13.35), versatiles were 34.03 (13.84), and tops were 35.47 (13.21). Racial self-identification was as follows: 301 participants classified themselves as White, 1 Black, 2 Chinese, 1 Filipino, 2 Aboriginal, 3 Latin American, 3 Southeast Asian, 1 West Asian, 2 Korean, 1 Japanese, 15 indicated “other,” and 1 participant declined to answer.

### Measures

*Sexual orientation* was defined by both self-identification (i.e., straight, gay, bisexual or other) and sexual attraction to the opposite- (i.e., straight) or same-sex (i.e., gay) in the last year. Specifically, participants were asked the gender(s) of individuals they were sexually attracted to in the last twelve months (similar to [22]). Responses were limited to a 5-point scale: *1) only females*, *never to males*, to *5) only males*, *never females*. An option was also available for those who identified as asexual–“*I have never engaged in sexual behaviour*.*”* Examples of sexual experience were given in parentheses “(*e*.*g*., *petting*, *kissing*, *oral sex*, *intercourse*).” Men who identified as straight and indicated an attraction to females only and never to males in the last year were categorized as straight. Men who identified as gay and indicated they were only attracted to males, never to females, in the last year were categorized as gay. All others who were not concordant for sexual attraction in the last year and their self-identified sexual orientation were excluded from analyses. In total, 39 participants were excluded: 14 were straight men (all of whom stated *“more often [attracted] to females but at least once to a male”*) and 25 were gay men (all of whom stated *“more often [attracted] to males but at least once to a female”)*. These predominantly gay or straight men were excluded because of recent evidence suggesting that mostly heterosexual individuals differ from exclusively heterosexual individuals on correlates of sexual orientation (for review, see [23]), and gay participants were defined similarly to be consistent across categories.

*Anal sex role preference* and *anal sex role behavior* were self-labeled by participants. Specifically, individuals who identified as gay men were asked whether they engage in and/or fantasize about anal sex. If the participant answered “yes,” they were asked (1) which role they prefer to take when engaging in and/or fantasizing about anal sex (i.e., preference), and (2) which role they usually take when engaging in anal sex (i.e., behavior). For both questions, their response options included “top,” “versatile,” or “bottom.” For the second question (regarding which role they usually take), they were also given the option to select *“Not sufficient experience with anal sex to answer*.*”*

*Handedness* was assessed using the 10-item Edinburgh inventory (writing, throwing, toothbrush, spoon, drawing, scissors, knife [without fork], striking a match, opening a box [lid], broom [upper hand]). Answers were provided on a 5-point likert scale: *1-always right* to *5-always left*. Scores for each of the 10 items were summed, which resulted in scores ranging from 10 (i.e., always use the right hand) to 50 (i.e., always use the left hand).

*Gender nonconformity* in the present study was assessed using the Recalled Childhood Gender Nonconformity Scale [24]. This 23-item questionnaire is scored such that high scores are male-typical and, therefore, low scores are gender-nonconforming. We present the data for a subset of the scale (18 items belonging to Factor 1 as described in [24]). This scale included questions such as “*As a child*, *my best or closest friend was 1-always a boy*, *to 5-always a girl*”, and “*In fantasy or pretend play*, *I took the role 1-only of boys or men*, *to 5-only of girls or women*”

### Statistical analyses

Gay and straight men were compared on age, Recalled Childhood Gender Nonconformity and Handedness using independent sample *t*-tests. One-way analyses of variance (ANOVAs) were used for the following group comparisons on Age, Handedness and Recalled Childhood Gender Nonconformity scale: (1) straight men and gay men categorized by anal sex role preference, and (2) straight men and gay men categorized by anal sex role behavior. As reported below, anal sex role preference groups did not differ on handedness, whereas anal sex role behavior group differences on handedness were found. Thus, further analyses compared anal sex role behavior groups, rather than preference groups. Furthermore, bottom and versatile anal sex role behavior groups were collapsed for analyses because these two groups were homogenous with respect to the focal variables of Handedness and Recalled Childhood Gender Nonconformity (Cohen’s *d* for handedness = 0.05 and for gender nonconformity = 0.04).

Following Baron and Kenny [25], we evaluated whether handedness mediated the relationship between gender nonconformity and sexual orientation—unstandardized coefficients (*B*) are reported. Next, we evaluated whether handedness mediated the relationship between gender nonconformity and anal sex role behavior. Lastly, we observed a curvilinear relationship between handedness and gender nonconformity (described below), such that the relationship ran in opposite directions for gay and straight men, and therefore we performed moderated mediation for handedness on the relationship between gender nonconformity among gay and straight men (i.e., Handedness X Sexual Orientation—details below). Sobel’s tests were conducted for all mediation analyses and one-tailed *p*-values were used given that the direction of the effect being tested was predicted *a priori* and known based on the results of the mediation analysis. Fisher’s Least Significant Difference (LSD) tests were used to examine pair-wise group differences in cases where the omnibus ANOVA indicated a significant overall effect. Significance was set to α = .05.

## Results and discussion

### Age

No difference in age was found between gay and straight men, *t*(331) = 0.349, *p* = .727. Similarly, no differences in age were found between preferred anal sex role groups of gay men and straight men based on preference, *F*(3, 329) = .104, *p* = .958, nor when based on behavior, *F*(3, 309) = .877, *p* = .453.

### Sexual orientation and Handedness

Descriptive statistics for the handedness scale of gay and straight men, as well as anal sex role preference and anal sex role behavior subgroups, are shown in Table 1. Consistent with previous research, gay men were less right-handed compared to straight men, *t*(331) = 2.319, *p* = .021.

**Table 1:** Descriptive statistics for the Handedness scale.

	*n*	Mean	SD
Straight Men	91	15.02	6.27
All Gay Men ^a^	242	17.14	9.83
Anal Sex Role Preference Groups:			
Tops	36	16.11	9.23
Versatiles	115	16.30	8.31
Bottoms	91	18.59	11.62
Anal Sex Role Behavior Groups:			
Tops	57	14.21	6.68
Versatiles	69	17.33	10.41
Bottoms	96	17.85	10.27
Bottoms/Versatiles combined ^a^^,^^b^	165	17.64	10.30

Note: Handedness scores ranged from 10 (i.e., always use right hand) to 50 (i.e., always use left hand). Bottom and versatile anal sex role behavior groups were collapsed for subsequent analyses because these groups were homogeneous with respect to the focal variables of the study, handedness and gender nonconformity.

^a^ Significantly different from straight men, *p* < .05.

^b^ Significantly different from gay men from the top anal sex role behavior group, *p* < .05.

### Anal sex role and Handedness

A one-way ANOVA evaluating Handedness between straight men and gay men categorized based on their anal sex preferences was not significant, *F*(3, 329) = 2.094, *p* = .125.

A significant effect was found between straight men and anal sex behavior groups on the Handedness scale, *F*(3, 313) = 4.575, *p* = .011. Posthoc analyses indicated that gay men who reported bottom/versatile anal sex as their typical sexual behavior were less right-handed compared to straight men and top gay men, *p* = .022 and *p* = .011, respectively. Top gay men did not differ from straight gay men, *p* = .581.

### Gender nonconformity

Descriptive statistics for the Recalled Childhood Gender Nonconformity scale of gay and straight men, as well as anal sex role preference and anal sex role behavior subgroups, are shown in Table 2.

**Table 2:** Descriptive statistics for the Recalled Childhood Gender Nonconformity scale.

	*n*	Mean	SD
Straight Men	91	4.34	0.39
All Gay Men ^a^	242	3.82	0.56
Anal Sex Role Preference Groups:			
Tops ^a^	36	3.98	0.50
Versatiles ^a^	115	3.83	0.61
Bottoms ^a^^,^ ^b^	91	3.75	0.50
Anal Sex Role Behavior Groups:			
Tops ^a^	57	3.96	0.52
Versatiles ^a^^,^ ^c^	69	3.80	0.59
Bottoms ^a^	96	3.78	0.57
Bottoms/Versatiles combined ^a^^,^ ^c^	165	3.79	0.57

Note: Lower scores indicate more gender nonconformity among males. Bottom and versatile anal sex role behavior groups were collapsed for subsequent analyses because these groups were homogeneous with respect to the focal variables of the study—handedness and gender nonconformity.

^a^ Significantly different from straight men, *p* < .05.

^b^ Significantly different from gay men with a top anal sex role preference, *p* < .05.

^c^ Significantly different from gay men with a top anal sex role behavior, *p* < .05.

Gay men were significantly more gender-nonconforming on the Recalled Childhood Gender Nonconformity scale compared to straight men, *t*(331) = 9.492, *p* < .001.

Anal sex role preference groups significantly differed on the Recalled Childhood Gender Nonconformity scale, *F*(3, 329) = 23.910, *p* < .001. Posthoc tests indicated that preferred bottoms/versatiles were significantly more gender-nonconforming compared to tops, *p* = .048, and straight men, *p* < .001. Preferred tops were also more gender-nonconforming compared to straight men, *p* < .001.

Similarly, anal sex role behavior groups significantly differed on the Recalled Childhood Gender Nonconformity scale, *F*(3, 309) = 22.429, *p* < .001. Bottoms/versatiles were more gender-nonconforming compared to tops, *p* = .032, and straight men, *p* < .001. Tops were also more gender-nonconforming compared to straight men, *p* < .001.

### Correlation analyses

**Fig 1:**
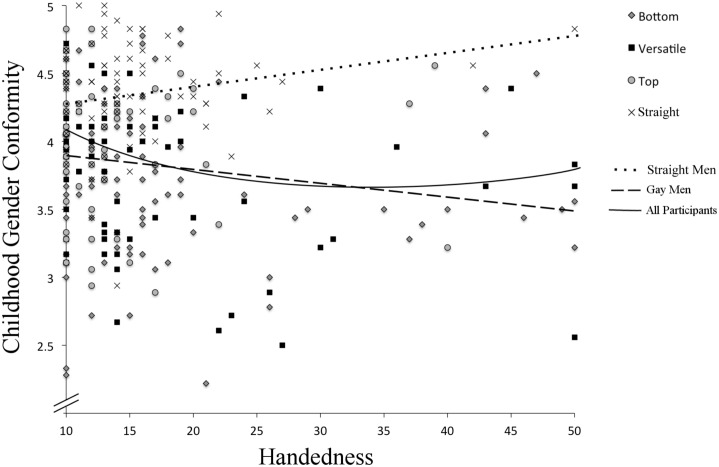
Curvilinear relationship between Handedness and Recalled Childhood Gender Nonconformity. A significant curvilinear relationship was found between Handedness and Recalled Childhood Gender Nonconformity such that participants with low and high handedness scores were more gender-conforming compared to participants with mixed handedness/intermediate handedness scores. Anal sex role preference groups are plotted as follows: circles represent top gay men, squares are versatile gay men, diamonds are bottom gay men and ‘x’ represent straight men. The solid line depicts the curvilinear relationship, whereas the dotted line represents the linear association for straight men, and the dash line represents the linear association for gay men.

Among gay men, a significant negative correlation was found between Handedness and Recalled Childhood Gender Nonconformity scores, *r*(242) = -.175, *p* = .006, indicating that increased non-right-handedness is associated with more gender nonconformity. Among straight men, these variables showed a marginally significant positive correlation that was nevertheless similar in magnitude (although notably opposite in direction) to the correlation found for gay men, *r*(91) = .19, *p* = .072. Although the correlation for straight men did not reach significance, the effect size suggests that this effect is of the same magnitude as that among gay men; thus, with a larger sample size, it is likely that this correlation would emerge as significant [for a review discussing the importance of considering both statistical significant and effect size, see 26]. Coupled with the findings reported above regarding male sexual orientation differences for these variables, these results suggest that there is a nonlinear relationship between gender nonconformity and handedness across the entire sample. Indeed, a significant curvilinear relationship was found, *R*^2^ (331) = .028, *p* = .009, such that participants with low and high handedness scores were more gender conforming compared to participants with mixed handedness/intermediate handedness scores (see Fig 1). This quadratic model accounted for more variance than the linear model, *R*^2^ (332) = .024, *p* = .003. Furthermore, the sample size composition from the present study is comprised of a majority of gay men (70.6%), thus it would be predicted that the magnitude of difference between quadratic and linear models would increase as the sample approached a sample comprised of 50% gay and 50% straight men.

### Moderated mediation analyses

**Fig 2:**
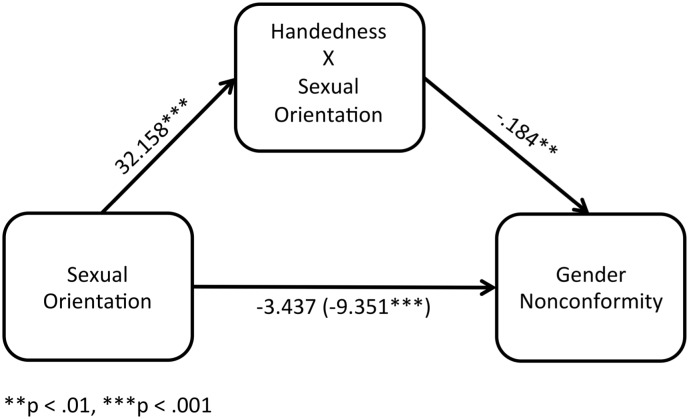
Moderated mediation of the Handedness by sexual orientation interaction on the male sexual orientation difference in Recalled Childhood Gender Nonconformity. A significant moderated mediation effect was found for Handedness on the male Sexual Orientation difference in Recalled Childhood Gender Nonconformity.

The correlations between Recalled Childhood Gender Nonconformity and Handedness scores indicated that the association between these variables runs in opposite directions for gay vs. straight men. As such, straight men’s Handedness scores were multiplied by -1 to create an appropriate interaction term to test for the moderated mediation effect of Handedness on the Sexual Orientation difference in Recalled Childhood Gender Nonconformity. The straight males were the reference group (see Fig 2).

In step 1 of the mediation model, the regression of the Handedness interaction term onto Sexual Orientation was significant, *B* = 32.158, *t*(331) = 29.041, *p* < .001. For the second step, the regression of Recalled Childhood Gender Nonconformity onto Sexual Orientation was also significant, *B* = -9.351, *t*(331) = -8.115, *p* < .001. For the final regression, the regression of Recalled Childhood Gender Nonconformity onto Sexual Orientation was no longer significant when the Handedness interaction term was also included in the model, *B* = -3.437, *t*(330) = -1.606, *p* = .109. Handedness significantly predicted Recalled Childhood Gender Nonconformity in this last step, *B* = -.184, *t*(330) = -3.262, *p* = .001. A Sobel’s test indicated a significant moderated mediation effect of Handedness on the male Sexual Orientation difference in Recalled Childhood Gender Nonconformity, *Sobel’s z* = -3.265, one-tailed *p* < .001.

**Fig 3:**
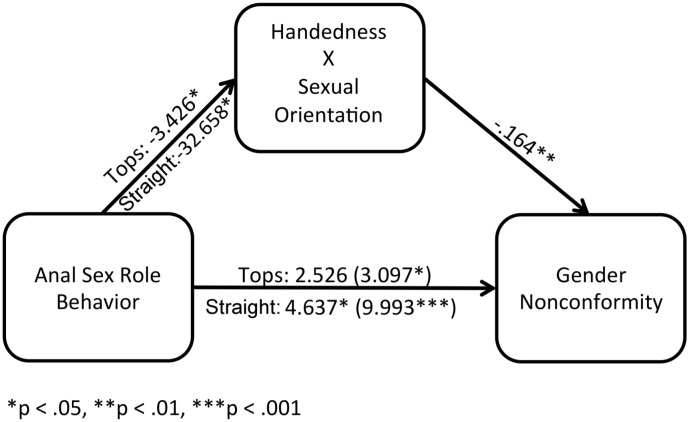
Moderated mediation of the Handedness by sexual orientation interaction on the anal sex role behavior difference in Recalled Childhood Gender Nonconformity. Bottom/versatile groups were used as the reference group. Results support a partial moderated mediation effect for Handedness on the anal sex role behavior difference in Recalled Childhood Gender Nonconformity.

Handedness was evaluated as a moderated mediator for Recalled Childhood Gender Nonconformity differences among anal sex role behavior groups. We used anal sex role behavior rather than anal sex role preference for these analyses because (as reported above) group differences in Handedness reached the critical alpha of < .05 when compared between anal sex role behavior, but not preference, groups. Again, straight males’ Handedness scores were multiplied by -1, but this time bottoms/versatiles were the reference group because they differed from tops and straight men on Handedness whereas these latter groups showed no such difference (See Fig 3).

In step 1 of the moderated mediation model, the regressions of Handedness onto anal sex role behavior groups were significant: straight men, *B* = -32.658, *t*(310) = -28.772, *p* < .001 and tops, *B* = -3.426, *t*(310) = -2.565, *p* = .011. For the second step, the regressions of Gender Nonconformity onto anal sex role behavior groups were significant: straight men, *B* = 9.993, *t*(310) = 8.216, *p* < .001, and tops, *B* = 3.097, *t*(310) = 2.157, *p* = .032. For the final regressions, with Handedness included in the model, straight men, *B* = 4.637, *t*(309) = 2.011, *p* = .045, and tops, *B* = 2.526, *t*(309) = 1.764, *p* = .079, were less significantly different on Recalled Childhood Gender Nonconformity compared to bottoms/versatiles, suggesting partial moderated mediation. Handedness significantly predicted Recalled Childhood Gender Nonconformity in this last step, *B* = -.164, *t*(309) = -2.722, *p* = .007. The Sobel’s test was significant for straight men, *Sobel’s z* = 2.721, one-tailed *p* = .003, and tops, *Sobel’s z* = 1.87, one-tailed *p* = .035. These findings again suggest a moderated mediation effect of Handedness on Sexual Orientation differences in Recalled Childhood Gender Nonconformity, and also Recalled Childhood Gender Nonconformity differences among subgroups of gay men delineated by anal sex role behavior.

## Conclusions

Consistent with previous research, male sexual orientation and anal sex role were associated with Recalled Childhood Gender Nonconformity scale scores in our sample. Gay men were more gender-nonconforming compared to straight men and gay men with a bottom/versatile anal sex role were more gender-nonconforming compared to both gay men with a top anal sex role and straight men. We also replicated the finding that gay men are more non-right-handed compared to straight men. We evaluated handedness among gay men grouped by their anal sex roles, and found that bottoms/versatiles were more non-right-handed compared to tops and straight men. Of particular importance, the present study suggests that differences in gender nonconformity between straight and gay men and among anal sex role subgroups of gay men are influenced by prenatal mechanisms linked to handedness.

Across both gay and straight men, a curvilinear relationship was found between handedness and gender (non)conformity. High (left) and low (right) handedness scores were associated with more recalled childhood gender conformity relative to participants with mixed/intermediate handedness scores; this pattern appeared to occur because the association between gender conformity and non-right-handedness is positive among straight men and negative among gay men (see Fig 1). Furthermore, moderated mediation analyses indicated that handedness mediated the overall male sexual orientation difference as well as anal sex role subgroup differences in gender (non)conformity (see Figs 2 and 3). To our knowledge, these are the first results to suggest that developmental processes related to handedness explain variation in gender expression both between straight and gay men as well as within these groups.

The biological mechanisms underlying handedness are not entirely clear. One prominent hypothesis has been that the sex difference is related to sex differences in prenatal testosterone [2]. If this hypothesis holds true, it would correspond well with the neurohormonal theory [27, 28] of sexual orientation development, which postulates that the prenatal surge in testosterone promotes male-typical brain development, giving rise to male-typical behaviors, including male-typical gender patterns and sexual partner preference (i.e., a preference for women). In males, female-typical gender expression and same-sex attraction are thought to result from a relative lack of prenatal testosterone exposure, resulting in female-typical brain development and, thus, female-typical behavior and sexual partner preference (i.e., a preference for men).

Evidence suggests that higher levels of prenatal testosterone are associated with more male-typical play patterns in early childhood in boys and girls [29, 30]. In addition, a putative marker of prenatal testosterone exposure (i.e., second-to-fourth digit ratio; for review, see [31]) was found to be more female-typical in males with a female gender identity, again suggesting a relationship between low testosterone and female-typical gender development [32]. The evidence-base is further strengthened by studies examining samples of individuals affected by disorders of sex development for which there is relative certainty regarding the prenatal sex hormone milieu. For example, female-typical development and gender identity is common among XY-individuals with androgen insensitivity syndrome, a condition that limits the action of androgens [33]. Likewise, females with conditions associated with elevated prenatal androgens such as congenital adrenal hyperplasia display more male-typical gender patterns than females not affected by the condition [34].

Findings relating prenatal testosterone to male sexual orientation or to handedness have been more mixed. For male sexual orientation, bio-correlates of prenatal testosterone have been found to be more female-typical (e.g., size of the third interstitial nucleus of the anterior hypothalamus: [35]), more male-typical (e.g., penis size: [36]) or not different from straight men (e.g., second-to-fourth digit ratio: [37]). Similarly, non-right-handedness has been linked to high prenatal testosterone (e.g., [38–40]) and low prenatal testosterone [41–43], while others did not find a relationship between testosterone and handedness [44, 45]. Interestingly, a recent study found evidence for a curvilinear relationship between handedness and markers of androgens, and between markers of androgens and androphilic attraction; quadratic effects are not frequently evaluated, which may account for the inconsistencies in this literature [46]. Specifically, Ellis, Skorska, and Bogaert [46] found: (a) a curvilinear relationship between handedness and markers of androgen in women, and a linear relationship between these variables in men (although the authors suggest that the relationship appears curvilinear for men as well, but the linear model, not the quadratic, was significant), and (b) for both men and women a quadratic relationship between androphilic attraction and markers of androgen.

Variation in androgen sensitivity is another possible explanation for these inconsistent findings (e.g., [47]). The X-chromosome linked androgen receptor gene (*AR*) has been associated with handedness [47]. Handedness has been associated with the polymorphic number of polyglutamine (CAG) repeats on the *AR* gene. Individual differences in CAG repeat lengths have been associated with the efficiency of the receptor such that *AR* variants with longer CAG repeat tracts are thought to function less efficiently (e.g., [48]).

Medland et al., [49] found that longer repeats were associated with left-handedness in females, and that shorter repeats were associated with left-handedness in males. This latter finding suggests that left-handedness is associated with more efficient AR in males and, thus, dovetails with the present data on straight men. Among straight men, a tendency toward non-right-handedness was associated with greater male-typical childhood behavior, possibly due to greater prenatal action of testosterone on brain masculinization.

Conversely, mixed handedness (i.e., the use of right hand for some tasks and left for others) was associated with longer CAG repeats in males in the study by Hampson and Sankar [43]. Arning et al., [47] found similar results: longer CAG-repeats were associated with mixed handedness in both men and women. These findings suggest that mixed handedness is associated with less efficient AR and, thus, dovetails with the present data on gay men, particularly those with a bottom or versatile anal sex role. It may be that this subset of men is particularly likely to have less efficient AR, leading to both non-right-handedness and more female-typical gender expression and sexual partner preference.

In sum, *AR* appears to be associated with handedness such that relative sensitivity of AR to testosterone might account for variation in handedness. If such is the case, then it is possible that subgroups of gay men delineated by handedness may differ from each other and from straight men in terms of their sensitivity to androgens. Interestingly, sexual orientation has been associated with other X-chromosome loci such as Xq28 (for review, see [50, 51]), although no link has yet been reported with regard to male sexual orientation and AR CAG repeat length [52]. Further research is needed to understand the individual and additive or interactive contributions of endocrine and genetic mechanisms underlying handedness, and this research may indicate these mechanisms as also underlying sexual orientation development.

These mechanisms may also be relevant to heterogeneity among gay men with respect to sexual behavior. Here, handedness significantly differed among anal sex role behavior groups (i.e., tops were more likely to be right-handed), and notably not among anal sex role preference groups. These patterns suggest that handedness is somehow associated with one or more behavioral mechanisms that influence whether a gay man is likely to perform a bottom/versatile vs. top anal sex role. Given that the handedness differences among anal sex role subgroups of gay men corresponded with parallel differences in recalled childhood gender (non)conformity (i.e., tops reported greater gender conformity), gender expression may be one such behavioral mechanism. Indeed, masculinity and stereotypes surrounding masculinity appear to influence decisions around anal sex role identity and sexual positioning practices among men who have sex with men [53]. As such, mechanisms linked with handedness may have an indirect influence on sexual behaviors such as anal sex role via their effects on gender expression, which is putatively important for influencing decisions about sexual positioning.

Although the precise contributions of genetic and hormonal processes to handedness and their relation to sexual orientation remain unclear, it should be noted that it is unlikely that these processes interact with mechanisms related to the fraternal birth order effect (i.e., the well-replicated finding that each additional older brother increases the odds of same-sex sexual attraction among males; [54]). This effect interacts with handedness such that only right-handed gay men exhibit it, whereas mixed- or left-handed gay men do not [16, 55– 58]. Bogaert [56] suggested that extremely right-handed gay men also do not show the fraternal birth order effect—only moderately right-handed gay men showed the effect. Furthermore, two additional studies did not find an association between the fraternal birth order effect and gender nonconformity [59, 60], whereas handedness mediated variation in gender nonconformity in the present study. Thus, it appears that mechanisms linking male sexual orientation to handedness differ from those linking it to the fraternal birth order effect (for review, see [61]).

In addition to the genetic and hormonal theories for the development of handedness, others have proposed that handedness and sexual orientation may be related to developmental instability [17]. Traits related to developmental instability such as minor physical anomalies and fluctuating asymmetry are more prominent in left-handers and extreme right-handers [62], and sexual orientation is associated with left-handedness and extreme right-handedness [17, 55, 56, 57, 58]. Although two studies [63, 64] did not find support for increased fluctuating asymmetry in gay men, both Miller, Hoffmann, and Mustanski [65] and Hall and Schaeff [66] did find increased fluctuating asymmetries, such as asymmetries in the ear, elbow, knee, ankle and finger length, among gay men compared to straight men. It is possible that developmental instability affects sexual differentiation of the brain and behavior and, thus, might also account for subgroups of gay men who differ in terms of gender nonconformity and/or anal sex role. This hypothesis could be tested by evaluating traits associated with developmental instability (e.g., minor physical anomalies, fluctuating asymmetry) in relation to sexual orientation, gender expression, and anal sex role. If this hypothesis is accurate, one would predict—based on patterns found in the present study—that gay men who are more non-right-handed, gender nonconforming, and bottom/versatiles would show increased traits of developmental instability.

### Limitations and future directions

The present study found that handedness mediates the relationship between male sexual orientation and childhood gender nonconformity. Thus, sexual differentiation mechanisms or mechanisms related to developmental instability that influence handedness may also regulate same-sex sexual orientation and gender nonconformity. It would be of interest, therefore, to evaluate the relationship between the prenatal endocrine environment and the AR gene (CAG repeats/sensitivity of the receptor) in relation to handedness, sexual orientation, anal sex role, and gender nonconformity. Although similar studies have been conducted on some of these variables individually or in pairs (e.g., handedness and AR [47], sexual orientation and handedness [16], anal sex role and gender nonconformity [19]), the present study suggests that this line of questioning merits a more comprehensive evaluation of these variables together, and that nonlinear relationships should be considered. It would also be of interest to investigate whether these relationships hold true for females and female same-sex sexual orientation.

Extreme right-handedness has been related to both male sexual orientation [16, 56] and increased gender nonconformity among gay men [58]. The sample size of the present study was insufficient to evaluate extreme right-handedness in relation to anal sex role groups and gender nonconformity. However, given that bottoms/versatiles were more non-right-handed than tops in the present study, it would be interesting to test whether bottoms/versatiles and tops similarly show differences in extreme right-handedness. These lines of investigation will help shed light on the mechanisms underlying sexual orientation, sexual behaviors such as anal sex role, and gender-related traits, as well as the developmental associations among these sexually differentiated traits in humans.

The present findings regarding gender nonconformity are consistent with previous reports suggesting that gay men who adopt a receptive anal sex role display more gender atypicality [19, 20, 21]. Men can experience erotic pleasure when their prostate gland is stimulated via receptive anal sex, and it is not uncommon for masculine straight men to enjoy being anally penetrated by a female partner through the use of objects such as a strap-on dildo [67]. Thus, findings suggesting that gay men who adopt bottom anal sex roles are more gender nonconforming do not necessarily warrant the interpretation that adopting a receptive anal sex role is itself a feminine trait.

The present study also does not necessarily provide any clear indication of how anal sex role might relate to handedness and/or gender nonconformity among straight men. Indeed, whereas non-right-handed gay men were more likely to be gender nonconforming and bottoms/versatiles behaviorally, non-right-handed straight men were more gender conforming. Given that the handedness and gender expression associations ran in opposite directions for gay vs. straight men, whether handedness and gender expression would map onto anal sex role behavior in a similar fashion among members of both groups is equivocal. Addressing this topic could be the focus of future research.

### Summary

The present study found that handedness, a sexually differentiated trait, mediates the relationship between sexual orientation and gender nonconformity, suggesting that the prenatal mechanisms underlying handedness also influence male same-sex sexual orientation and gender nonconforming behaviors. Furthermore, the present findings suggest that the biological mechanisms that underlie handedness differentially apply to subgroups of gay men delineated by their anal sex role behavior. Lastly, a curvilinear relationship was found between handedness and gender nonconformity, suggesting that high and low handedness scores are associated with more gender conformity compared to participants with intermediate/mixed handedness. Together, these findings suggest that the developmental processes underlying handedness act in a nonlinear fashion to influence male same-sex sexual orientation and childhood gender nonconformity. Lastly, the observed differences in handedness and gender expression among subgroups of gay men delineated by anal sex roles support the notion that gay men constitute a heterogeneous group with respect to developmental underpinnings of their same-sex sexual orientation.
